# Research on a Magnetic Separation-Based Rapid Nucleic Acid Extraction System and Its Detection Applications

**DOI:** 10.3390/bios13100903

**Published:** 2023-09-23

**Authors:** Yao Li, Sha Liu, Yuanyuan Wang, Yue Wang, Song Li, Nongyue He, Yan Deng, Zhu Chen

**Affiliations:** 1 Hunan Key Laboratory of Biomedical Nanomaterials and Devices, Hunan University of Technology, Zhuzhou 412007, China; liyao_666@163.com (Y.L.); lsdd0789@163.com (S.L.); w3568970551@163.com (Y.W.); yuesir0029@163.com (Y.W.); sosong1980@gmail.com (S.L.); nyhe@seu.edu.cn (N.H.); hndengyan@126.com (Y.D.); 2 State Key Laboratory of Digital Medical Engineering, School of Biological and Medical Engineering, Southeast University, Nanjing 210096, China

**Keywords:** nucleic acid extraction, magnetic separation, automated system, detection

## Abstract

Nucleic acid extraction represents the “first step” in molecular diagnostic experiments. The quality of this extraction serves as a fundamental prerequisite for ensuring the accuracy of nucleic acid detection. This article presents a comprehensive design scheme for a rapid automated nucleic acid extraction system based on magnetic separation. The design and implementation of the system are analyzed and investigated in-depth, focusing on the core methods, hardware control, and software control of the automated nucleic acid extraction system. Additionally, a study and evaluation were carried out concerning the nucleic acid extraction and detection aspects encompassed by the system. The results demonstrate that the temperature deviation in the lysis and elution fluids is approximately ±1 °C, the positioning accuracy of the system’s movement is ±0.005 mm, the average magnetic bead recovery rate is 94.98%, and the average nucleic acid recovery rate is 91.83%. The developed automated system and manual methods are employed for sample extraction, enabling the isolation of highly pure nucleic acids from bacteria, blood, and animal tissues for RT-PCR detection. The instrument employs lysis temperatures ranging from 70–80 °C, elution temperature of 80 °C, and drying time of 5–10 min, with a total extraction time of less than 35 min for different sample types. Overall, the system yields high nucleic acid concentration and purity, exhibits stable instrument operation, good repeatability, high efficiency, and low cost. It meets the requirements of genetic-level research and is worthy of clinical promotion and usage.

## 1. Introduction

Nucleic acids, composed of multiple nucleotides, are essential biomacromolecules in biological systems [1,2,3]. They can be classified into deoxyribonucleic acid (DNA) and ribonucleic acid (RNA). Extraction of DNA and RNA from biological samples constitutes a crucial step in molecular biology and molecular diagnostics [4,5,6]. It serves as the foundation for subsequent experiments and aims to isolate and purify the target molecules from biological samples, enabling their utilization in various experimental procedures such as polymerase chain reaction (PCR) amplification, sequence analysis, gene expression profiling, and mutation detection [7,8,9,10,11]. The success of these downstream experiments greatly relies on the quality of the extracted nucleic acids, with the sensitivity and specificity of the extraction method playing a pivotal role.

The process of nucleic acid extraction comprises sample preparation, extraction, and purification [12,13,14,15]. Traditional extraction methods include boiling lysis, phenol–chloroform extraction, high salt precipitation, alkali extraction, silica column method, magnetic bead method, and ion exchange method [16,17,18,19,20,21,22]. Each of these methods has its advantages and disadvantages (Table 1). Automated nucleic acid extraction instruments have the potential to improve workflow and reduce variability in clinical laboratories. Some reports have already identified the value of automated nucleic acid extraction instruments compared to manual methods [23,24,25,26]. Although most reports mention the benefits of streamlining workflows and reducing hands-on time, the quality of extracted nucleic acids can vary. 

Common automated nucleic acid extraction methods include those based on silica or silicon membrane columns, microfluidic chips, and magnetic separation. Among these, the silica-based method utilizes materials such as silica gel or silica columns to adsorb nucleic acids, followed by different washing and elution steps for nucleic acid purification [27,28,29]. Examples of this method include the Promega Maxwell series and the Zymo Research Quick-DNA/RNA series. These methods offer high levels of nucleic acid purity and are suitable for processing large batches of samples. However, they involve relatively complex operations and are not easily automated to a high degree. Microfluidic chips represent a miniaturized experimental platform that controls liquid flow through tiny channels and valves to capture and purify nucleic acids using chemical, physical, or biological methods [30,31,32,33]. Examples of products using this technology include the Qiagen QIAcube Connect MDx and Labcyte Echo 525 Liquid Handler. These are suitable for microvolume samples and can handle batch processing. However, microfluidic chips can be costly, and their use is limited by the channel size, making them suitable for specific sample types. The magnetic bead method is based on the efficient capture and separation of nucleic acid molecules using magnetic particles. Magnetic beads typically have ligands on their surfaces that are specific to nucleic acids such as DNA or RNA. After modification (e.g., surface modification with silica or carboxylation), they can selectively bind to nucleic acid molecules [34,35,36,37,38,39]. Examples of products using this approach include the Thermo Kingfisher series and Roche MagNA Pure LC. These systems allow for high-throughput and rapid nucleic acid extraction and are adaptable to various sample types, but they come with higher costs and require dedicated magnetic separation equipment and bead reagents.

Additionally, there are various fully automated nucleic acid extraction instruments available in the market, each accompanied by their corresponding nucleic acid extraction reagents [40,41,42]. It is important to note that different fully automated nucleic acid extraction platforms may yield varying extraction results. Nationally and internationally, nucleic acid extraction methods commonly face issues such as limited contamination control measures, relatively long extraction times, and complex, less user-friendly human–machine interfaces [43,44]. These challenges restrict the applicability of the instruments and make them less suitable for on-site nucleic acid extraction. The selection of these methods and instruments should be based on specific requirements, sample types, and laboratory conditions to ensure the best nucleic acid extraction results.

Based on the aforementioned challenges, considering the high purity, high yield, and fast processing speed offered by automated nucleic acid extraction, we have developed a high-performance and personalized magnetic bead-based fully automated nucleic acid extraction system (magnetic rod-based). This system addresses various aspects, including magnetic separation structure, motion structure, heating structure, and overall system design. We have successfully demonstrated its effectiveness in DNA extraction and qPCR using diverse biological samples, such as bacteria, animal tissues, and blood. Through adjustments, optimizations, and upgrades of instrument parameters based on different biological sample tests, we have further improved the system’s sensitivity and reduced errors, thus enhancing the accuracy of the extraction process. This development will actively contribute to the advancement of the nation’s healthcare industry, which is a key focus area for national development.

## 2. Materials and Methods

### 2.1. Structural Design

**Figure 1:**
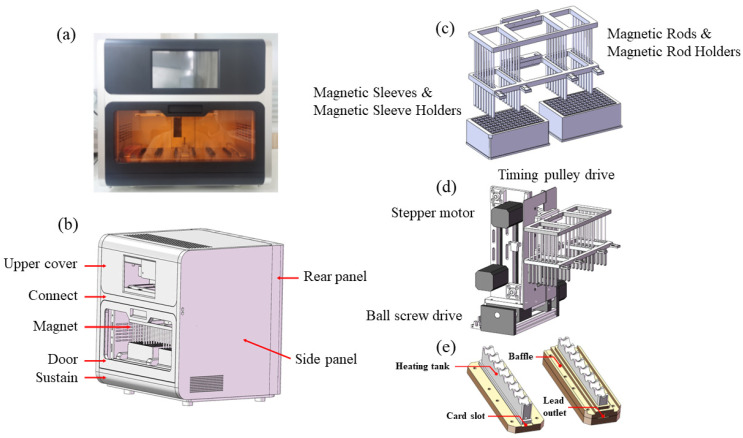
(**a**) Front view of the nucleic acid extraction instrument; (**b**) Design schematic of the nucleic acid extraction instrument’s exterior; (**c**) Magnetic rod-based separation module; (**d**) Mechanical drive module; (**e**) Heating trough structure.

Figure 1a,b illustrate the schematic diagram of the fully automated nucleic acid extraction system. The critical components of this system comprise three parts: the magnetic separation module (Figure 1c), the motion module (Figure 1d), and the heating module (Figure 1e). The magnetic separation module consists of a magnetic sleeve, magnetic rod, magnetic rod holder, and magnetic rod rack. To prevent liquid splashing, the system processes a maximum sample volume of 1 mL, and we selected magnetic sleeves (Beijing Baiter Biotechnology Co., Ltd., Beijing, China) with dimensions of 45 mm in height and 6 mm in diameter, designing the magnetic sleeve holder accordingly. The motion module employs a stepper motor-driven transmission structure with dual-thread linear guides as guiding components, allowing precise positioning and movement of the magnetic separation structure within the nucleic acid extraction device to complete the entire extraction process. The ball screw drive is actuated by a stepper motor via a coupling, which in turn drives the horizontal movement of the sliding table panel fixed on the screw rod. The motor drives the driving wheel to rotate, thereby driving the driven wheel on the other end of the synchronous belt to rotate, achieving the vertical movement of the magnetic separation structure. The X-axis provides precise horizontal positioning of the entire system at various positions in the deep-well plate, while the Y-axis enables the magnetic separation structure within the entire system to perform corresponding experimental operations at different positions in the deep-well plate. For the heating module design, we created a heating trough with dimensions of 74 mm in length and 7 mm in width to better fit the bottom of the microplate. The flexible and adaptable Polyimide (PI) heating film was utilized for effective heat transfer. The material used for the baffle is Polyether Ether Ketone (Peek), which possesses high-temperature resistance and corrosion resistance. The overall design of the instrument’s structure takes into consideration the feasibility and stability of the entire system’s operation while meeting both user aesthetic preferences and functional requirements of the system in a three-dimensional design.

### 2.2. Hardware Design

**Figure 2:**
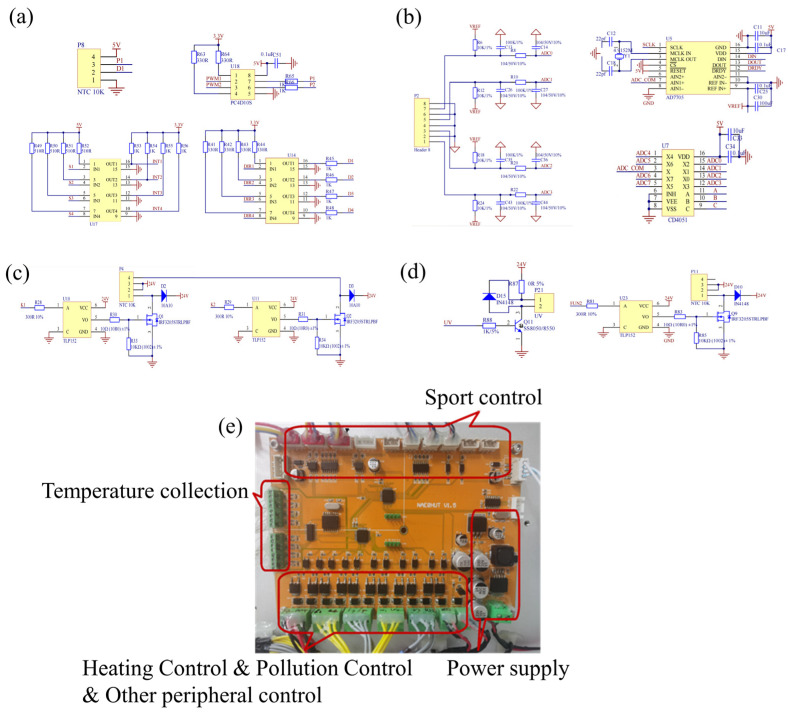
(**a**) Motion control module schematic design; (**b**) Temperature control module acquisition circuit schematic design; (**c**) Temperature control module heating circuit schematic design; (**d**) Anti-pollution control module schematic design; (**e**) Circuit board physical picture.

The hardware design of this system utilizes a master-slave dual-chip (STM32F103C8T6, ST) control scheme. The main microcontroller unit (MCU) is responsible for controlling the motion control module, taking into account factors such as accuracy, speed, stability, and cost. The PKP246D23A model stepper motor is chosen, along with the MD-2322 driver module from ShanShe and the optoelectronic sensors of the EE-SX671-WR series [45,46,47]. By implementing hardware circuit design for the motion module as shown in Figure 2a, applying certain pulse signals to the PWM1 and PWM2 pins, the PC4D10S chip performs the corresponding level conversion and outputs pulse signals based on the corresponding level, determining the movement distance of the motion control module. The pulse signal frequency determines the rotational speed of the stepper motor. Nonlinear optocouplers TLP521-4 are used for both U14 and U17. The former controls the direction of the stepper motor, while the latter enables limit and zero-point positioning control of the stepper motor through the use of photodetection. The Sub-MCU Controller primarily controls the temperature module within the system. The temperature control module utilizes an NTC10K thermistor as the temperature sensor. The circuit design includes a data acquisition circuit (Figure 2b) for collecting real-time data from the temperature sensor and a heating circuit (Figure 2c) for controlling the heating element. The CD4051 and AD7705 chips are employed, along with a reference voltage VEF, a pull-up 10K resistor divider, and a multiplexer switch for temperature selection. The temperature is converted into a digital signal by the A/D conversion chip and processed by the MCU. The TLP152 chip in U10 serves to isolate the input and output circuits. K1 and K2 are pulse-width modulation waves controlled by the MCU, connected to the input pins of the TLP152 chip. After the output voltage drives Q1, controlling the output voltage of P4, they regulate the heating rate of the heating trough. The combination of these two components forms a feedback loop for precise control of the heating temperature. To prevent cross-contamination in the system, a contamination control module has been implemented, as depicted in Figure 2d, incorporating ultraviolet disinfection and the HAPE system.

The MCU and other functional modules are integrated into the circuit board, constituting the lower-level components of the nucleic acid extraction system. The physical assembly of the circuit board, shown in Figure 2e, includes the power supply section, motion control components, temperature acquisition unit, heating control, contamination control, and other peripheral control sections.

### 2.3. Software Design

**Figure 3:**
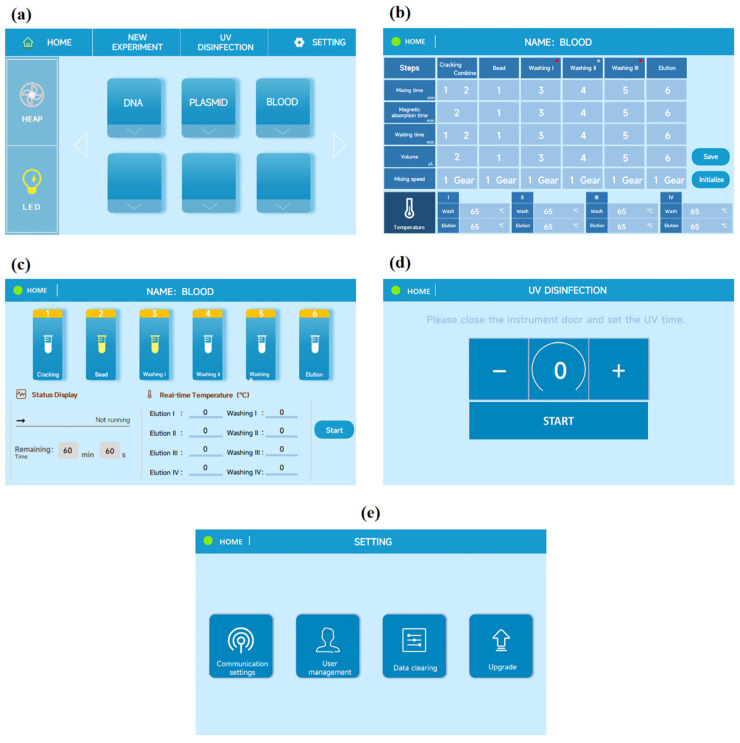
(**a**) Main interface design diagram; (**b**) Program editing interface design diagram; (**c**) Program running interface design diagram; (**d**) UV disinfection interface design diagram; (**e**) System setting interface design diagram.

Software development entails the development of both lower-level firmware and upper-level user interfaces for human–machine interaction. The lower-level firmware development involves embedded programming of the STM32F103C8T6 microcontroller chip, using the Keil5 development environment to create a program for automating the nucleic acid extraction process. Temperature control is achieved using the Proportional-Integral-Derivative (PID) algorithm, which regulates the STM32F103C8T6 chip on the control circuit board. This allows the timer to generate a pulse-width modulation (PWM) waveform with an adjustable duty cycle, enabling precise control of the current to the heating element. Serial communication is employed between the microcontroller and the touch screen (Beijing DWIN Technology Co., Ltd., Beijing, China) to establish a communication protocol between the lower-level controller and the upper-level software, thereby achieving automation of nucleic acid extraction. The primary focus of the lower-level development is to facilitate effective human–machine interaction. The user interface comprises five key sections. The Section 1 is the main interface (Figure 3a), which enables users to directly select options for air filtration lighting and adjust the built-in experimental parameters based on specific requirements. The Section 2 is the program editing interface (Figure 3b), allowing users to set the required experimental parameters during the extraction process, such as step durations, temperatures, rotational speeds, and volumes of various reagents. Click the red dot to clear the value in this column. The Section 3 is the program execution interface (Figure 3c), which provides a real-time display of the experiment’s status, remaining time, and temperature during the extraction process. The Section 4 is the UV disinfection interface (Figure 3d), employed for UV disinfection of the instrument’s interior before or after experiments to effectively mitigate batch contamination. The Section 5 is the system settings interface (Figure 3e), providing users with the capability to update and upgrade the instrument’s software, perform communication calibration, and meet diverse user requirements and preferences effectively.

### 2.4. DNA Extraction and RT-PCR Detection

**Figure 4:**
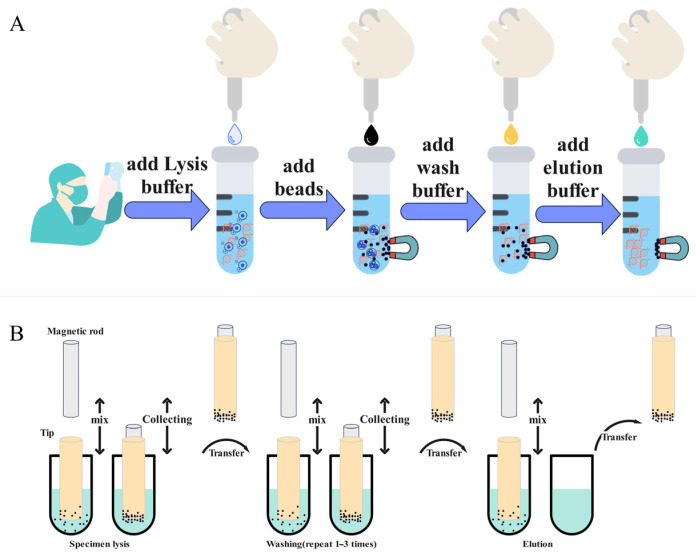
(**A**) Manual nucleic acid extraction by magnetic bead method; (**B**) Automatic nucleic acid extraction by magnetic bead method.

The Pseudomonas aeruginosa strain (ATCC9027) required for the experiment is sourced from the Zhuzhou Hospital, an affiliate of the Xiangya School of Medicine at Central South University. It is cultured and provided by the laboratory or testing department at this institution. Mouse liver tissue and blood samples were obtained from residual specimens from previous experiments [48], and comprehensive details regarding animal welfare and ethical considerations as previously described were provided. A commercially available magnetic bead-based nucleic acid extraction kit was uniformly employed for the extraction process. Specifically, the 32T Magnetic Bead Bacterial Nucleic Acid Extraction Kit (Zhuhai BaoRui Biotechnology Co., Ltd., Zhuhai, China) was used. For manual extraction (Figure 4A), 200 μL of Pseudomonas aeruginosa cultured overnight in a carbon dioxide incubator (Shanghai Lishen HF151) is added to a centrifuge tube, followed by the addition of 10 μL of proteinase K, 10 μL of magnetic bead solution, and 600 μL of lysis buffer LB (containing Tris-HCl buffer, NaCl, EDTA, etc.). The mixture was vigorously shaken and incubated at 65 °C in a metal bath for 5 min. Subsequently, the samples were washed with 600 μL of wash solution W1 and then with wash solution W2 (containing ethanol, surfactant Tween 20, etc.). After discarding the supernatant, the sample was air-dried for 10 min, followed by the addition of 100 μL of elution buffer EB (containing Tris-EDTA buffer, ddH_2_O, etc.) and elution at 85 °C for 10 min. For automated extraction (Figure 4B), 200 μL of the sample to be extracted, 600 μL of lysis buffer LB, and 10 μL of proteinase K were added to well 1 of the instrument’s accompanying 96-well deep plate. Well 2 received 10 μL of magnetic bead solution, well 3 contained 600 μL of wash solution W1, well 4 contained 600 μL of wash solution W2, and well 6 contained 100 μL of elution buffer EB. The lysis temperature was set to 80 °C for 5 min with a wait time of 10 min, followed by elution at 80 °C for 10 min.

The 32T Magnetic Bead Tissue Nucleic Acid Extraction Kit (Zhuhai BaoRui Biotechnology Co., Ltd.) was utilized. Take a 10 mg tissue block and grind it thoroughly. Add 200 μL of pre-processing solution to it. Centrifuge at 12,000× *g* rpm for 3 min, and then collect the supernatant. For manual extraction, 200 μL of the sample was mixed with 600 μL of lysis buffer LB and 50 μL of magnetic bead suspension. The mixture was incubated at 55 °C in a metal bath for 10 min. Following this, a single wash step with 600 μL of wash solution W1 and two wash steps with 600 μL of wash solution W2 were performed. After discarding the supernatant, the sample was air-dried for 10 min, and then 100 μL of elution buffer EB was added for elution at 60 °C for 10 min. For automated extraction, 200 μL of the sample to be extracted, 600 μL of lysis buffer LB, and 20 μL of proteinase K were added to well 1 of the accompanying 96-well deep plate. Well 2 received 50 μL of magnetic bead solution, well 3 contained 600 μL of wash solution W1, well 4 contained 600 μL of wash solution W2, well 5 contained 600 μL of wash solution W2, and well 6 contained 100 μL of elution buffer EB. The lysis temperature was set to 70 °C for 10 min, with a waiting time of 5 min, followed by elution at 80 °C for 10 min.

The Magnetic Bead-Based Blood Nucleic Acid Extraction Kit (Luoyang Aisen Biotechnology Co., Ltd., Luoyang, China) was employed. For manual extraction, 200 μL of the sample was mixed with 20 μL of lysis buffer B and incubated at 75 °C in a metal bath for 5 min. Subsequently, 600 μL of magnetic bead solution and 300 μL of binding solution A were added, followed by another incubation at 75 °C for 5 min. Afterward, two washes with 600 μL of washing solution were conducted. Finally, the supernatant was discarded, and the sample was air-dried for 10 min. Subsequently, 100 μL of elution buffer was added, and the elution was carried out at 75 °C for 10 min. For automated extraction, 200 μL of the sample to be extracted, 20 μL of lysis buffer B, and 300 μL of binding solution A were added to well 1 of the accompanying 96-well deep plate. Well 2 received 600 μL of magnetic bead solution, well 3 contained 600 μL of wash solution, well 4 contained 600 μL of wash solution, and well 6 contained 100 μL of elution buffer EB. The lysis temperature was set to 80 °C for 10 min, with a waiting time of 10 min, followed by elution at 80 °C for 10 min.

The extracted nucleic acid solution was characterized by agarose gel electrophoresis. Weigh 0.125 g of agarose powder and measure 25 mL of 1× TAE buffer using a graduated cylinder to prepare a 0.5% agarose gel. Add it into a special conical flask for electrophoresis, shake it gently to mix, place it in a microwave oven, and heat it at medium-high temperature for 2 min until the mixture is completely dissolved. Add 1.5 μL of ethidium bromide (EB) solution to the mixture and pour it into a gel tray. Let it solidify at room temperature for approximately 30 min. After the gel has solidified, remove it from the gel tray using a comb and immerse it in an electrophoresis chamber to prepare for sample loading. Take 5 μL of the extracted material and mix it evenly with 1 μL of loading buffer. For this experiment, use a DNA marker with sizes ranging from 500 to 15,000 bp. Pipette 5 μL of the DNA marker into one of the gel wells. Adjust the voltage to 150 V and the current to 120 mA, then run the electrophoresis for approximately 30 min.

Target amplification was performed using real-time fluorescent quantitative PCR (qPCR) detection. The Pseudomonas aeruginosa nucleic acid detection kit (Guangdong HuanKai Biotechnology Co., Ltd., Guangzhou, China), murine-sourced nucleic acid detection kit, and universal nucleic acid detection kit (Guangzhou HuaFeng Biotechnology Co., Ltd., Guangzhou, China) were employed. In a reaction volume of 25 μL, a cycling program was applied to different nucleic acid extraction solutions as follows: For the Pseudomonas aeruginosa samples, 5 μL of nucleic acid extraction solution was subjected to an initial denaturation cycle at 95 °C for 30 s, followed by 40 amplification cycles. Each cycle consisted of denaturation at 95 °C for 5 s and annealing at 60 °C for 40 s.

Regarding murine liver tissue samples, 2.5 μL of nucleic acid extraction solution underwent an initial denaturation cycle at 90 °C for 1 min, followed by 40 amplification cycles. Each cycle involved denaturation at 95 °C for 10 s and annealing at 60 °C for 30 s.

For 3 μL of human blood nucleic acid extraction solution, a single cycle was performed at 55 °C for 5 min, followed by another cycle at 95 °C for 5 min, and then a repeating cycle of 10 s at 95 °C, 30 s at 60 °C, and 40 s at 72 °C, repeated 40 times. All qPCR reactions were meticulously conducted under consistent and specified conditions using the designated Real-Time PCR instrument (Thermo Fisher Scientific ABI 7500, Waltham, MA, USA).

## 3. Results and Discussions

### 3.1. Performance Index Evaluation of Automatic Extractor

**Figure 5:**
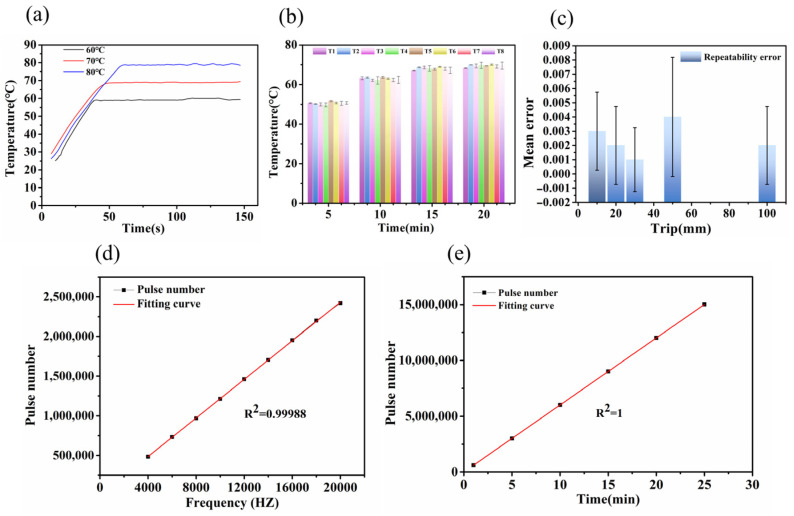
(**a**) Temperature curve control chart of heating tank; (**b**) Liquid temperature values of 8 holes; (**c**) Repeatability error under different strokes; (**d**) Number of pulses achieved under different frequencies; (**e**) Under different times.

During the nucleic acid extraction process, the lysis temperature is typically maintained within the range of 55–75 °C, while the elution temperature is usually set between 60–80 °C. To ensure minimal variations in nucleic acid purity and concentration, we conducted temperature testing for both the lysis and elution steps. Firstly, we evaluated the performance of the heating block by setting it at three different temperatures: 60 °C, 70 °C, and 80 °C. As shown in Figure 5a, the heating module reached a stable preset temperature within approximately 49–52 s, achieving complete temperature control stability within 75 s. The temperature control accuracy was within ±0.5 °C, demonstrating its suitability for the intended application. Secondly, we assessed the liquid temperature within the deep well plate. To represent the sample throughput of our system (32-sample capacity), eight measurement points were evenly selected and labeled as T1, T2, T3, T4, T5, T6, T7, and T8. The deep well plate was subjected to a 20 min heating process, during which real-time temperature data was collected using a data logger (KSB08A0R), as depicted in Figure 5b. The temperature of the liquid in the deep well plate can reach 60 °C in 10 min and 65 °C in 15 min. The temperature differences between individual wells were within approximately ±1 °C.

To ensure that the magnetic sleeve and magnetic rod remain positioned directly above the center of the deep well plate throughout their movement, high precision is required in driving the stepper motor. In this study, we measured the repeatability error of the stepper motor after 50 cycles of back-and-forth movement at different travel distances of 20, 40, 60, 80, and 100 mm. The experiment was conducted using a grating ruler, with the error read by a digital indicator connected to the grating ruler. Figure 5c shows the repeatability error, which was within ±0.005 mm, meeting the requirements of most applications. It indicates that the stepper motor’s accuracy is sufficient for precise positioning. Oscillation mixing is essential for controlling the vertical movement of the magnetic sleeve during nucleic acid extraction. It is a crucial step in the nucleic acid extraction process. At the end of the mixing process, we measured the error between the final position of the magnetic rod and its initial position. Figure 5d represents the number of pulses generated at different frequencies (The oscillation speed can be adjusted between 1 and 9 levels). A linear fit was observed with a coefficient of determination of 99.99%. The results demonstrate that the effect of frequency on travel distance is negligible and can be disregarded. Figure 5e illustrates the number of pulses generated at different time intervals during the mixing process. A strong linear relationship with a coefficient of determination of 100% was observed. This indicates that there is minimal positional accuracy error and excellent repeatability during the mixing process.

**Figure 6:**
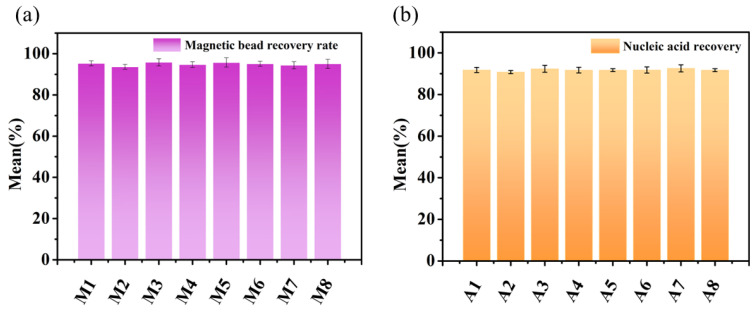
(**a**) Magnetic bead recovery; (**b**) Nucleic acid recovery.

The recovery rate of magnetic beads is one of the crucial factors influencing the efficiency of nucleic acid extraction. Therefore, we calculated the magnetic bead recovery rate by weighing the mass of the beads before and after extraction. Eight measurement points, labeled as M1–M8, were selected. We performed three measurements of 50 μL of magnetic bead solution, averaged the results, and obtained a value of 2.98 mg as the initial magnetic bead mass before transfer. In the deep well plate, 600 μL of deionized water was sequentially added to the lysis, magnetic bead activation, wash I, wash II, wash III, and elution wells. A 50 μL volume of the magnetic bead solution was added to well 2. After the experiment, the beads were dried and weighed to obtain the final bead mass. Figure 6a shows the magnetic bead recovery rates at the eight measurement points after three repeated experiments. The range of magnetic bead recovery rates was 93.6–95.83%, with a standard deviation between 1 and 2.5 and a coefficient of variation <3%. The overall average magnetic bead recovery rate for the system was determined to be 94.98%.

Improving the nucleic acid recovery rate is helpful for enhancing experimental efficiency and accuracy. To validate the accuracy and reliability of our nucleic acid extraction system, we employed spectrophotometry using salmon sperm DNA as the sample (initial concentration: 1024 ng/μL). Prior to extraction, the sample was subjected to spectrophotometric analysis to determine the initial nucleic acid concentration. Based on the number of wells used for extraction in the system (32-sample throughput), eight measurement points (labeled as A1–A8) were evenly selected for testing. Following the instructions provided with the nucleic acid extraction kit, 10 μL of salmon sperm DNA solution was used. The solution was sequentially added to the wells: 15 μL of magnetic bead solution +400 μL of MA solution in the magnetic bead activation well, 700 μL of 70% ethanol solution in the wash I and wash II wells, and 100 μL of TE solution in the elution well. After the experiment, the nucleic acid concentrations in the eight centrifuge tubes were determined using a spectrophotometer. Figure 6b shows the nucleic acid recovery rates at the eight measurement points after three repeated experiments. The range of nucleic acid recovery rates was 90.8–92.59%, with a standard deviation between 0.7 and 1.7 and a coefficient of variation <2%. The overall average nucleic acid recovery rate for the system was determined to be 91.83%.

### 3.2. Comparisons in Different Samples

**Figure 7:**
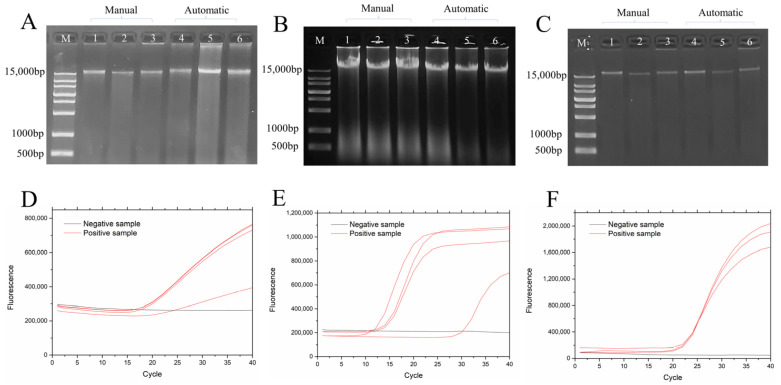
(**A**) Pseudomonas aeruginosa nucleic acid extraction electrophoresis results; (**B**) mouse liver tissue nucleic acid extraction electrophoresis results; (**C**) human blood nucleic acid extraction electrophoresis results; (**D**) Pseudomonas aeruginosa PCR amplification results; (**E**) mouse PCR amplification results; (**F**) Whole blood PCR amplification results.

Nucleic acid extraction was performed on Pseudomonas aeruginosa samples, mouse liver tissue, and human blood using both manual and automated methods, as shown in Figure 7A–C. The manual method required operation times of 54 min and 50 s, 49 min and 30 s, and 44 min and 40 s, respectively. In contrast, the automated instrument completed the process in 33 min, 30 min, and 28 min for the corresponding samples. The nucleic acid extraction system we developed yields nucleic acid concentrations comparable to the manual method but significantly reduces the extraction time. It eliminates complex operations, making it well-suited for high-throughput and rapid extraction requirements. To remove RNA contamination, an appropriate amount of RNA degrading enzyme was added, resulting in an OD_260_/OD_280_ ratio ranging from 1.8 to 2.0.

According to the specifications of the reagent kit, in Figure 7D–F, the positive samples used were automatically extracted products. During the amplification of Pseudomonas aeruginosa and mouse tissue, experiments were also conducted using the positive samples provided with the assay kit. When the Ct value for the test sample is ≤35, it indicates the presence of the target gene in the extracted components, demonstrating the validity of the experimental results. This suggests that the DNA extraction was effective, the PCR amplification was successful, and the sample DNA can be used for subsequent experiments.

### 3.3. Sensitivity Evaluation and Optimization

Taking into consideration the balance between reaction time and efficiency, we explored the optimization of magnetic bead washing time and drying time in the system. By optimizing the washing time, we found that extending the washing time to 2 min did not significantly improve the nucleic acid extraction concentration. We also set a gradient of drying times at 5 min, 10 min, and 15 min and observed that different samples required different drying times (Table 2). For example, when extracting mouse liver tissue as a sample, a drying time of only 5 min achieved a similar effect as a manual extraction with a 10 min drying time. Additionally, we evaluated the sample lysis and elution temperatures. The results showed that setting the instrument’s lysis temperature between 70 °C and 80 °C and the elution temperature at 80 °C was suitable. If the temperature is too low, the extraction efficiency decreases, while excessively high temperatures can cause magnetic beads to crack. These preliminary results demonstrate the feasibility of applying this automated nucleic acid extraction instrument to biological samples.

Compared to existing nucleic acid extraction instruments in the market, such as the SSNP-9600A from Shuoshi, which offers a 96-well throughput but lacks a user-friendly interface and the flexibility to modify experimental procedures, our system offers several significant advantages. Our system uses embedded technology to organically combine various functional modules, such as motion, temperature, human–computer interaction, and other modules. The stepper motor drives the synchronous wheel to drive the synchronous belt to achieve vertical movement and drives the ball screw to achieve horizontal movement with good repeatability. It employs an incremental PID algorithm to precisely control temperature, meeting practical temperature requirements. A more user-friendly human-computer interaction interface is designed, and the touch screen of Dacai Company is selected as the control panel. The operating interface and parameter setting interface are designed and displayed independently, which conforms to the user’s usage habits and makes the operation simpler and easier to use. Moreover, our system allows users to modify, delete, or customize experimental parameters to meet individualized requirements, providing greater flexibility. UV disinfection and air filtration systems have also been added to effectively prevent cross-contamination. In the extraction of real samples, the extraction time is greatly shortened, and it can be well suited for downstream applications.

## 4. Conclusions

In summary, this study has developed a rapid nucleic acid extraction system based on magnetic separation, which can be employed for DNA extraction and RT-PCR detection. It has demonstrated good extraction performance for bacteria, animal tissues, and blood samples. Compared to traditional manual extraction methods, automated nucleic acid extraction offers the ability to process a large number of samples rapidly. It also allows for better control of experimental parameters, reducing the risk of operational errors and sample contamination. This not only saves time and effort for laboratory personnel but also enhances the accuracy of the analytical results. Our automated system’s extraction instrument operates stably, achieves high extraction purity, and significantly reduces processing time. It exhibits similar extraction capabilities to commercially available instruments in terms of sensitivity, stability, and processability. It is compatible with DNA/RNA extraction commercial kits and can be used for downstream nucleic acid detection, facilitating clinical promotion and application.

## Figures and Tables

**Table 1:** Comparison of nucleic acid extraction methods.

Method	Purity	Stability	Skills Requirement	Hazardous Reagents	Time	Multi-Step Centrifugation	Automation
Phenol chlorine method	High	Low	High	Need	Longer	Need	No
High salt precipitation method	Low	Low	High	Unnecessary	Long	Need	No
silica-based column method	High	High	Low	Unnecessary	Short	Need	Yes
Ion exchange method	Higher	High	High	Unnecessary	Longer	Unnecessary	Yes
Magnetic separation method	Higher	High	Low	Unnecessary	Short	Unnecessary	Yes

**Table 2:** Extraction effects of Pseudomonas aeruginosa, mouse liver tissue, and blood samples at drying times of 5 min, 10 min, and 15 min, respectively.

Sample	Time (min)	OD_260_/OD_280_	OD_260_/OD_230_	C, ng/μL
Pseudomonas aeruginosa	5	1.94	1.85	148.7
	10	1.95	1.99	152.4
	15	1.88	2.01	155.8
Mouse liver tissue	5	2.01	2.03	974.1
	10	2.04	2.13	1004.6
	15	2.03	2.02	988.9
Blood	5	1.91	1.92	16.3
	10	1.94	2.00	12.5
	15	1.94	1.98	13.7

## Data Availability

Not applicable.
